# Binding of Different Cyclosporin Variants to Micelles Evidenced by NMR and MD Simulations

**DOI:** 10.3390/membranes13020196

**Published:** 2023-02-05

**Authors:** Polina P. Kobchikova, Sergey V. Efimov, Vladimir V. Klochkov

**Affiliations:** Institute of Physics, Kazan Federal University, 18 Kremlevskaya, 420008 Kazan, Russia

**Keywords:** cyclosporin, NMR, molecular dynamics, micelle

## Abstract

Peptides play a critical role in the life of organisms, performing completely different functions. The biological activity of some peptides, such as cyclosporins, can be determined by the degree of membrane permeability. Thus, it becomes important to study how the molecule interacts with lipid bilayers. Cyclosporins C, E, H and L were characterised molecular dynamics simulation; NMR spectroscopy studies were also carried out for cyclosporins C and E. The comparison of one- and two-dimensional spectra revealed certain similarities between spatial structures of the studied cyclosporin variants. Upon dissolving in water containing DPC micelles, which serve as model membranes, subtle changes in the NMR spectra appear, but in a different way for different cyclosporins. In order to understand whether observed changes are related to any structural modifications, simulation of the interaction of the peptide with the phospholipid micelle was performed. The onset of the interaction was observed, when the peptide is trapped to the surface of the micelle. Simulations of this kind are also of interest in the light of the well-known membrane permeability of cyclosporin, which is important for its biological action.

## 1. Introduction

Cyclosporins are ring-closed peptides isolated from fungi, having the molecular weight of 1.2 kDa. The most widely used is cyclosporin A. It has immunosuppressive properties, depending on the permeability of the cell membrane, since it needs to penetrate into the cell cytoplasm to form a complex with cyclophilin [1]. Besides immunosuppressive properties, cyclosporin A also shows antiviral and anti-inflammatory activity. Despite all the advantages, side effects are also observed, the main of which is neurotoxicity [2]. A number of other cyclosporins remain under close scrutiny of scientists in attempts to find or create such an analogue of cyclosporin A, which would have diminished side effects. For example, cyclosporin G demonstrated good results in animals, while simultaneously possessing immunosuppressive properties such as cyclosporin A and having the least pronounced nephrotoxicity, but, unfortunately, the results were not so obvious in humans [3,4]. Other analogues of cyclosporin, not possessing immunosuppressive properties, are also of pharmacological interest because they demonstrate potentially useful antiparasitic and antiviral effects [5,6].

The application of NMR techniques to proteins interacting with membranes, especially integral ones, is not straightforward. Solid-state techniques study the membrane proteins in the most natural environment, but suffer from limitations of spectral resolution and require special probes [7,8]. Micelles of surfactants or phospholipids used as a solubilising medium can also be used in studies of membrane-associated molecules, especially small-sized peptides. The choice of the medium will be a compromise considering several factors such as overall particle size, biocompatibility and the type of information required [9,10]. Various model membrane types have been invented so far: micelles, bicelles [11], lipid–protein nanodiscs [12,13], multilamellar vesicles made of POPC [14,15], etc.

Perdeuterated dodecylphosphocholine was specifically developed as a membrane-like detergent for use in NMR experiments. The predominant phospholipid in animal (but not bacterial) cell membranes, DPC is nevertheless a true detergent with properties very similar to SDS [16]. To date, DPC is one of the most thoroughly studied model systems for studying lipid-binding peptides and proteins. Examples of detergents used as a model membrane are numerous and include studies of antimicrobial peptides [17], mitochondrial 18-kDa translocator protein TSPO [18], lytic peptide melittin found in bee venom [19,20,21], etc.

As mentioned above, the pharmacological properties depend on the membrane permeability. Since a different polarity of the environment is observed outside and inside the cell membrane, and the conformation of the molecule may depend on the environment, the necessity of studying the structure of cyclosporin in polar and non-polar media arises. The conformations that cyclosporins can take in polar and non-polar media have been investigated in some studies [22,23,24,25,26]. Experimental investigations involving NMR spectroscopy, mass-spectrometry or membrane-permeation measurements offer insight into the averaged features of the molecules. Molecular dynamics provides information on faster processes, but should be corroborated by experimental data. In this work, we try to expand our understanding of the behaviour of cyclosporin on the lipid–water interface. In particular, we are interested in the onset of the binding of cyclosporin to the micelle, molecular flexibility and pattern of intramolecular hydrogen bonds present in the phospholipid environment. The structure of the molecules is considered on the qualitative level using NMR data.

## 2. Materials and Methods

**Figure 1:**
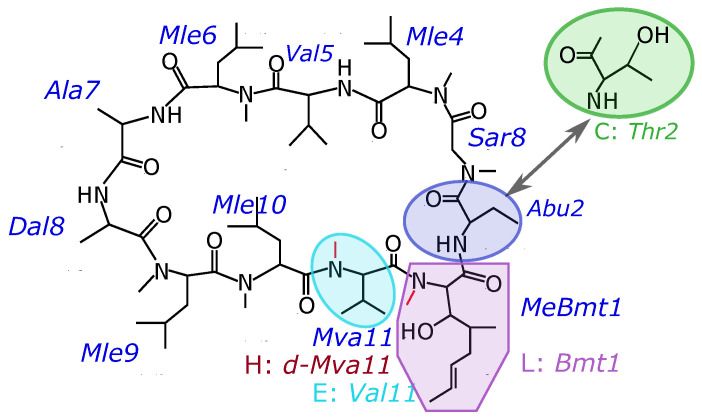
Chemical formulas of the studied variants of cyclosporins.

The composition of four peptides that were the objects of this study are presented in Table 1. In cyclosporin C (CsC), the second amino acid is replaced by threonine. For other cyclosporins studied, aminobutyric acid (Abu) is in the second position. In cyclosporin L, the first amino acid Bmt1 is non-methylated instead of the carrying NCH${}_{3}$ group, while in CsE the 11th amino acid (Val) is non-methylated. Cyclosporin H differs from CsA by the optical isomer of the residue Mva11. Chemical formulas are shown in Figure 1.

Samples for NMR experiments contained ∼1 mg peptide, giving the final concentration of about 1 mM, and 7–9 mg dodecylphosphocholine. Cyclosporin H was purchased from Cayman Chemicals; other peptides, from AvaChem Scientific; DPC, from Avanti Polar Lipids. Powders were mixed and dissolved in water. A mixture of 90% H${}_{2}$O and 10% D${}_{2}$O was used to observe amide proton signals, which exchange with the solvent within minutes; pure D${}_{2}$O (Solvex-D) was used for better quality of the Hα spectral region. A clear solution was obtained after stirring and sonication. The resulting concentration exceeds the critical micelle concentration for DPC (1 mM); furthermore, NOESY spectra demonstrated negative nuclear Overhauser effect and strong cross-peaks, which is the indication of the low mobility of the observed molecules, with the correlation time τ obeying the inequality τω≫1 for the spectrometer frequency $\omega =4.4\times 10^{9}$ rad/s. Note that in solvents such as chloroform or DMSO cyclosporin exhibits small and positive or small and negative NOE, which means that τω∼1. The effective molecular weight of the observed molecular system is much larger than 1.2 kDa, and hence it consists of peptide-surfactant aggregates.

Measurements were carried out on a Bruker Avance III HD 700 spectrometer in 5-mm tubes at 25 °C. The WATERGATE pulse sequence was used to suppress the water signal in samples dissolved in H${}_{2}$O/D${}_{2}$O. Two-dimensional NOESY spectra were obtained with the mixing time from 0.1 to 0.2 s.

MD simulations were carried out in GROMACS 2018.6 software. Molecular ensembles for the simulations were composed of a cyclosporin molecule and a micelle made of 54 unimers (downloaded from [27]; corresponding lipid force field parameters are described in [28]). A model of desired cyclosporin can be obtained in different ways, for example, adapting a model of cyclosporin A found in the PDB base. NCH${}_{3}$ group in Mva11 or Bmt1 can be easily replaced by a proton to obtain NH group in CsE or CsL, respectively. More complex modifications can be made, for example, with the aid of the Chimera programme [29] and its module SwissSidechain [30]. Initial peptide models were taken from other GROMACS trajectories simulated earlier in chloroform; as will be discussed below, the difference between structures in a low-polarity solvent and in complex with the micelle is not expected to be very prominent. Cyclosporin was added to be in the solvent near the micelle and partially overlap with phospholipid molecules; in the latter case, the DPC molecules overlapping with the peptide were removed (48–52 remained). Water (tip3p) was then added to the cubic simulation cell with the size from 5.0 to 5.7 nm. The system was subject to the steepest descent energy minimisation, followed by the NVT ensemle calculation for 1 million steps with dt=0.8 fs. PME method was employed to calculate electrostatic energy with the cut-off distance of 1.0 nm and the fourth order interpolation. NPT ensemble was used in the main part of the simulation with the same time step; Berendsen barostat was used; temperature was set to 298 K and pressure to 1 bar. Trajectory durations varied from 36 to 80 ns.

## 3. Results

### NMR Spectroscopy

The use of surfactants (including membrane-mimicking media) allows for dissolving cyclosporins in water in a concentration sufficient for NMR measurements. The average aggregation number of DPC micelles is 56, as stated in [9]. In the study of 18-residue antimicrobial peptide protegrin-1, the model of the micelle contained 60 DPC or SDS molecules [31]. Even larger micelles are possible, which contain 100 of unimers and more, but they appear at bigger surfactant concentrations and usually have a non-spherical shape [32]. Thus, if the molar concentration of the surfactant is ∼50 times larger than that of the peptide, one peptide molecule will be incorporated on average in one micelle, and we can avoid a possible interaction between the cyclosporin molecules. Signals of the surfactant in the high-field region of the spectrum overlap severely with the peptide’s signal under these conditions, so the low-field amide region remains the most convenient for analysis.

**Figure 2:**
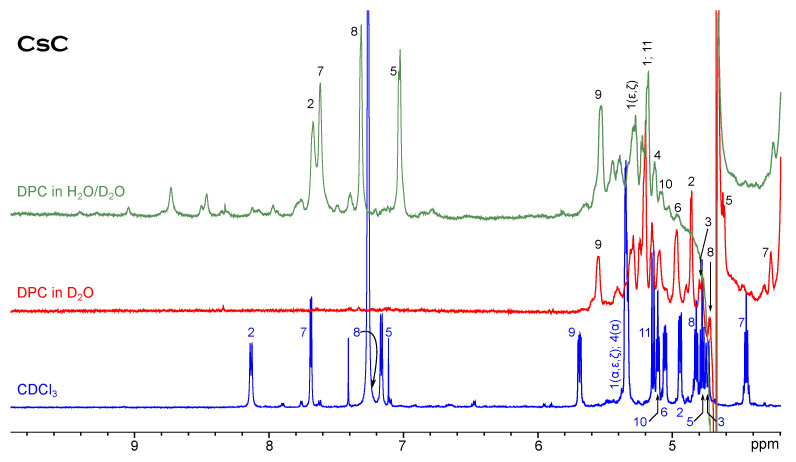
${}^{1}$H NMR spectrum of CsC in chloroform compared to spectra in micellar solution.

Figure 2 shows NH and Hα signals of cyclosporin C in CDCl${}_{3}$ and water/DPC mixture. Evidently, one conformer still remains dominant in the micellar solution; resonances are generally slightly shifted to the higher field. The extent to which the resonance positions change is not the same for different amino acids, but the order of signals does not alter too much. Only the peaks Dal8(NH) and Mva11(Hα) shift in the opposite direction. Another difference is the presence of minor conformer signals. At least two sets of minor signals can be distinguished from 7.3 to 9 ppm, and their relative intensity is larger than in the chloroform solution. Cyclosporin A shows a similar behaviour.

**Figure 3:**
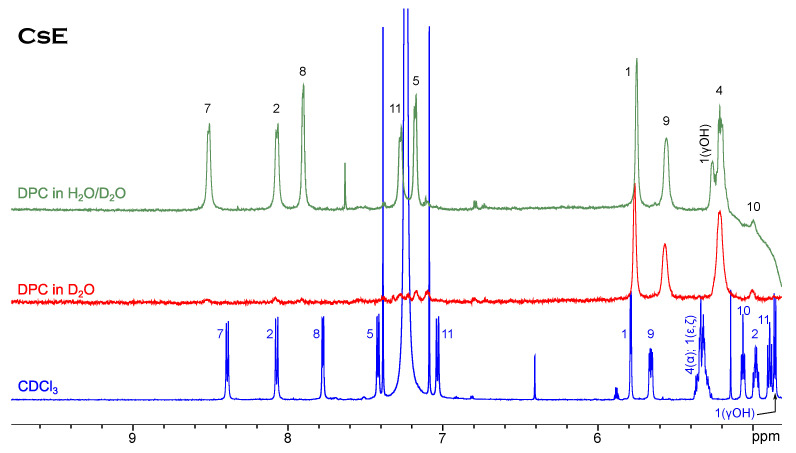
${}^{1}$H NMR spectrum of CsE in chloroform compared to spectra in micellar solution.

Ths case of cyclosporin E is slightly different (Figure 3). Neither in the chloroform solution nor in the CsE/DPC micelle complex can minor conformers be observed. The tendency to the upfield shift of resonances of the alpha-protons is still present, but among amide resonances only Val5 moves far to the right-hand side of the spectrum, while Dal8 and especially Val11 show a remarkable down-field shift.

Chemical shifts depend on several factors, including the conformation of the studied molecule, surrounding molecules and the presence of hydrogen bonds. Scalar coupling observed as the splitting of resonance lines is another source of information on the molecular geometry, which can be compared more or less directly with the structural data obtained by MD simulations. Therefore, we measured vicinal scalar couplings for amide protons from 1D spectra of cyclosporins in chloroform, where this splitting can be reliably observed. The obtained numbers are listed in Table 2. Calculated values in this table are obtained as described below in Discussion.

## 4. Molecular Dynamics

**Figure 4:**
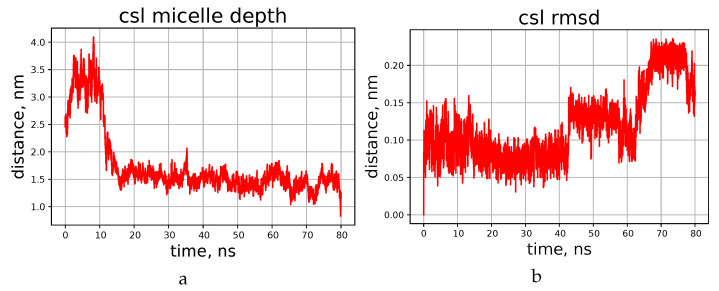
Time dependence of the distance from the peptide to the micelle’s center (**a**) and RMSD of the backbone atom positions (**b**), according to the simulations of cyclosporin CsL.

It turned out that during first few nanoseconds, the peptide ring lies onto the surface of the micelle flatwise, but does not penetrate deeper during the simulated period of time. This process is visualised in Figure 4a,b, which shows the time dependence of the distance between the centre of geometry of all lipid atoms present in the system and the center of geometry of all atoms of the peptide. The final distance (depth of penetration into the micelle) tends quickly to a certain limit, which is about 1.5 nm for cyclosporins L and E, and somewhat shorter for C and H.

Resulting peptide structures differ from those obtained in chloroform mainly by the orientation of the side chains, while the shape of the backbone is similar in both media. Amino acids comprising cyclosporin are hydrophobic, so their side chains can reside freely among DPC molecules. For example, the side chain of the residue Bmt1 is found oriented towards the centre of the micelle in CsC and CsE (see Supplementary Materials Figures S10 and S11); however, this is not necessary condition. The peptide molecule becomes partially immersed in the micelle in about 10 ns. This is shown on the example of cyclosporin L in Figure 4; corresponding plots for other peptides can be found in the Supplementary Information file, Figures S1 and S2.

To ensure that the cyclosporin did not remain on the micelle surface during the simulation, the depth to which it was immersed was determined. For this, the distance between the geometric centres of cyclosporin and micelle was compared with the distance from certain atoms of some selected DPC molecules to the centre of the entire micelle. Thus, it was determined that CsL stays at the level of the twelfth carbon of the DPC acyl chain, while the other cyclosporins studied (C, H, E) remained at the thirteenth carbon level during the simulation.

**Figure 5:**
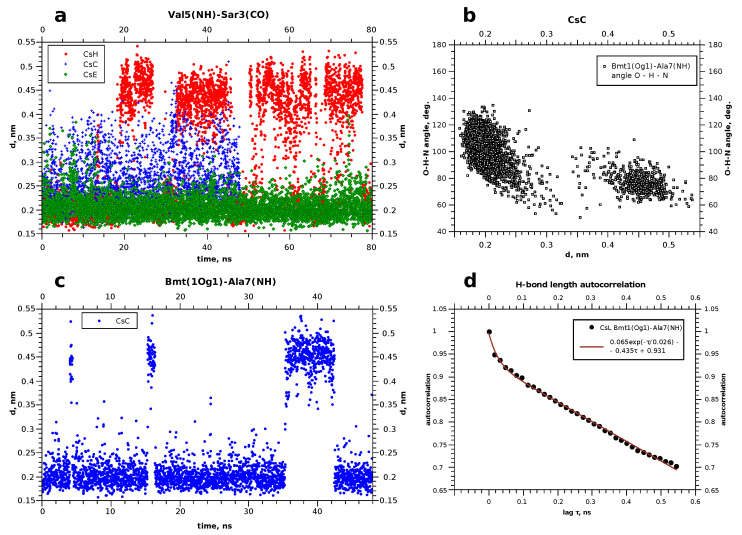
(**a**,**c**) Evolution of some hydrogen bonds in cyclosporins. (**b**) Correlation between the O–H distance and O–H–N angle in the case of bimodal behaviour, when the hydrogen bond disrupts and appears again. (**d**) Example of the autocorrelation for the hydrogen bond existance function.

Plots presented in Figure 5b,c show evolution of some atom-atom distances in H–O pairs capable of forming hydrogen bonds. More data of this kind, and also RMSD of backbone atoms during the simulation can be found in Supplementary Info (Supplementary Materials Figure S2 in addition to Figure 4 here, and also Supplementary Materials Figures S3–S5). Evidently, in this timescale the peptide can form and lose H-bonds in different chain segments from time to time, and the duration of existence of the “bonded” and “free” states can differ significantly. RMSD value of the backbone atom positions also experiences abrupt jumps, but stays at a constant level between the jumps. We interpret this as conformational transitions, which equilibrate quickly. Occasionally the RMSD level returns to one of the early met values, thus showing that there are several conformation types existing for tens of nanoseconds or even less. Continuous transitions between the states with similar energies can occur in different time scales [22,33,34], and quick local modifications observed here should be the phenomenon of the same kind. Interestingly, these modifications do not have clear correlation with the penetration depth achieved to the moment.

To reveal possible internal features, which are responsible for the existence of several conformers, we defined periods of time when different intramolecular hydrogen bonds were present. Several snapshots of the trajectory were taken at the moments, corresponding to different possible states visible in the RMSD plots. Hydrogen bonds were found in the peptide using Chimera [29], and then corresponding distances between in the O–H pairs were tracked using the MD trajectories.

This figure gives an example of the evolution of some hydrogen bonds. The bonds which occurred in all three cyclosporins were chosen. As can be observed, the distance in the atom pair Bmt1(Og1)–Ala7(H) in cyclosporin C is less than that of 0.25 nm for time periods of 10–15 nanoseconds, and increases occasionally to more than 0.4 nm. This is interpreted as a relatively stable H-bond. In general, in cyclosporin H, the hydrogen bonds are less stable. The most long-living and stable H-bonds are met in CsE, such as Val5(H)–Sar3(O), as shown in Figure 5a. Table 3 summarises changes in the hydrogen bonds over time.

## 5. Discussion

It is an admitted fact that cyclosporin can interact with phospholipid bilayers and membrane-mimicking media. Details of how this process occurs on the molecular level over time are hidden, and we have to unveil them using more or less indirect methods. We have revealed that the patterns of signals in the NOESY spectra of cyclosporin variants B, C, D and E, which reflect the close intramolecular contacts between protons and thus describe the molecular geometry, are nearly the same for these peptides in chloroform and in complex with DPC micelles [35]. For example, peptide bond Mle9–Mle10 is still in the *cis*-conformation, as proved by the Hα–Hα NOE contact (see the spectra in Supplementary Materials Figures S7 and S8). NOESY is a far more detailed method for characterising molecular structure than one-dimensional spectroscopy, and yet Figure 2 and Figure 3 show that molecules have different behaviours in two solvents used. The suggestion that cyclosporin E has a relatively rigid chain is corroborated by the absence of minor conformers in the micellar solution. Moreover, the hydroxyl proton Bmt1(OHγ) of CsE gives a separate signal in the DPC/H${}_{2}$O mixture, proving that it is firmly fixed by an intramolecular H-bond.

Vicinal couplings are more direct source of structural information than chemical shifts, though also prone to conformational exchange. Each cyclosporin contains four or five NH protons, and observed spectral splittings typically lie in the range from 6.5 to 8.6 Hz. Such a large value agrees with the elongated structure of the peptide ring, in which both sides form the structure resembling a β-sheet. Scalar couplings were calculated based on the phi angles to check which of these two states corresponds better to the NMR experiment [36]:

$${}^{3}J=6.51\cdot \cos^{2}\left(\alpha \mp 60^{\circ }\right)-1.76\cdot \cos\left(\phi \mp 60^{\circ }\right)+1.6\;.$$

Dihedral angles ϕ were tabulated from all saved snaphots of the MD trajectories. Scalar couplings were then calculated using the above formula for each snaphot, and finally averaged over the whole trajectory to give the calculated values in Table 2. As in the case of peptide variants studied so far (B, C, D) in chloroform, some dihedral angles ϕ of molecules in complex with DPC micelles experience fast jumps, and hence the final distribution of their values over all MD trajectory turns out to be bimodal (see Table 2). In this case, the observed ${}^{3}J$ value strongly depends on the populations of the local conformations, which cannot be established reliably in an accessible simulation time, and therefore the calculated and observed values can differ. The largest discrepancy is observed for the second residue in all cyclosporins. In other residues, however, calculations are generally consistent with the large values typical of the elongated ring shape.

Table 3 demonstrates that two hydrogen bonds occur in most cases. These are Bmt1(Hγ1)–Bmt1(O) and Val5(H)–Sar3(O). The former is found in all cyclosporins (in CsE, however, this distance experiences short but numerous jumps above 0.25 nm), and Val5(H)–Sar3(O) is found everywhere except CsL. The most numerous and stable hydrogen bonds are observed for cyclosporins C and L. CsH turned out to be the most unstable regarding the number and duration of existence of H-bonds.

Initially H-bonds were detected in snaphots of the peptide taken at different times. Then, corresponding distances between the oxygen and hydrogen atoms were traced to the obtained pictures such as shown in Figure 5a,c. Small values (<0.3 nm) were interpreted as the evidence of formation of the H-bond. This was also corroborated by the calculation of the N–H⋯O or O–H⋯O angles. Typically, the bond angle is about 150°; angles involving gamma-hydroxyl proton in threonine Bmt1 or Thr2 may be somewhat smaller but still obtuse (for example, see Figure 5c). This can be interpreted as the relative weakness of these bonds [37].

Finally, we can conclude that the bonding of cyclosporins to DPC micelles is proved not only by NMR methods, but is also observed by molecular dynamics’ methods and occurs in a typical time of 10 ns. Peptide penetrates in the layer under the polar groups and stays there. If it is able to go deeper in the micelle, remains unclear in the simulations performed. Examples of disposition of molecules in the cyclosporin-micelle complexes before and after the NPT simulation are shown in Supplementary Materials Figures S9–S11; Supplementary Materials Figure S9 illustrates also several intermediate states of the system.

The graphic in Figure 5 demonstrates local conformational changes which occur at different time scales: rapid jumps of the interatomic distances by several angstroms taking about 1 ns and slower changes of the conformation, when a certain state exists for tens of nanoseconds. This resembles the behaviour observed in simulations in CHCl${}_{3}$ [35]. Thus, we can notice that the phospholipid surrounding does not stop the internal dynamics of the peptide, as the dihedral angles and distance between atoms (including those forming hydrogen bonds) continue to evolve in time. However, altered solvent accessibility should lead to particular differences in the conformers’ arising. This suggestion is corroborated by the inequal changes in the chemical shifts of amide ${}^{1}$H and carboxyl ${}^{13}$C upon binding to the micelle, when most of the signals, but not all, remain nearly in the same positions. This is clearly observed for cyclosporins C and E and reflects changes in H-bonds pattern arising when the peptide has a different molecular environment (in our case, chloroform and the aqueous solution with model membranes).

Table 3 shows also some aspects of the time behaviour of the revealed hydrogen bonds. The periods when they exist or not can differ in duration significantly without a clear correlation with the relative populations of the states with and without the H-bond. The problem is that the periods when the corresponding H⋯O distance is short can be comparable with the obtained MD trajectory duration, and increase in the computational time is not an effective way to solve this problem. Thus, typical autocorrelation for the function showing the H-bond existence and absence in our case is a slowly descending function such as that plotted in Figure 5d. For this reason, we summarise in Table 3 the information on the relative fraction of all MD snapshots when an H-bond was observed, and also demonstrate the longest revealed existence period. Thus, cyclosporin C turned out to have H-bonds in five different sites, but Bmt1(Hγ1)–Val5(O) is the weakest one and most rare. Residues Dal8 and Val11 in CsE occur close to each other, and the H-bonds between them appears often but are relatively short-living (<10 ns). On the contrary, Val5 and Sar3 in CsE were always connected by the H-bond. It is worth noting that with the performed MD durations within 80 ns, we cannot evaluate accurately the relative lifetime of the H-bonds which were found partially stable; information about them should be rather considered qualitative.

Slow conformational changes caused by the *cis-trans* isomerism can also be influenced by binding to the membrane surface. For instance, this was observed for adrenocorticotropin hormone peptide and its fragments (from 10 to 32 residues): changes in the *cis-trans* equilibria occur upon binding to SDS and DPC micelles [38]. A similar phenomenon was observed for cyclosporin [33]; moreover, the ability to change the conformation is assumed to be the key property in the biological effect of this drug. In addition to “open” and “close” conformations, a more compact “bent” state of cyclosporin A has been revealed recently using ion-mobility spectrometry–mass spectrometry methods [34].

## 6. Conclusions

In this work we used molecular dynamics to observe how cyclosporin molecule binds to the DPC micelle in water solution when placed close enough to its surface (2–2.5 nm from the micelle centre in our simulations). Depending on the initial conditions, e.g., orientation of the peptide ring, there is no guarantee that the peptide will be caught by the micelle. Still peptide–micelle complex formation is quite probable; in our case, five out of six independent starts were successful.

Once formed, this model allows studying the structure of the molecule and its dynamics on the time scale of tens of nanoseconds. Phospholipid surrounding serves as a simple model of cell membrane in both computer simulations and NMR measurements. Cyclosporin variants differing by subtle variations in their chemical structure are known to have very different biological activities, including immunosuppression. There may be many reasons for this, and interaction with cell membrane is among the most actively discussed.

Cyclosporins E and L differ from most other variants, including CsA, by the presence of an additional (fifth) amide proton in positions Bmt1 and Val11, respectively. CsC has four NH protons but an additional (second) OH proton in the side chain Thr2. One can expect that this can alter the pattern of intramolecular hydrogen bonds. Table 3 proves this suggestion, showing that different H-bonds tend to arise in different peptides. Does this lead to significant consequencies for the peptide–membrane interaction? The changes in the overall shape of the peptide ring are subtle. For CsC and CsE, this is additionally proved by NOESY cross-peaks (Supplementary Materials Figures S5 and S6). The interplay between the apolar core of the micelle and water outside may be responsible for the resulting depth of penetration of the peptide under the surface. This parameter was found to be also similar for the studied compounds. A further membrane permeation for these peptides should require conformation change and is a far slower process.

Cyclosporin E, unlike most other variants, is conformationally pure in solution. The relative backbone rigidity of CsE, established earlier from the absence of minor conformers in NMR spectra recorded in CDCl${}_{3}$ and by MD simulations, is also observed in a complex with phospholipid micelles, which may be one of the reasons for the diminished interaction of CsE with a mitochondrial pore complex revealed in [35]. However, this rigidity is not so absolute in the CsE–DPC system, as follows from the time behaviour of interatomic distances in the middle of the simulation (15–48 ns). Evidently, binding to the micelle causes a perturbation, which leads to breaking of some hydrogen bonds, and then the peptide molecule reaches equilibrium again.

## Figures and Tables

**Table 1:** Composition of cyclosporins. Short names for N-methylated amino acids: Mle for N(Me)Leu and Mva for N(Me)Val. Dal is dAla.

Cyclosporin	Amino Acid Sequence	Chemical Formula	CAS No.
CsC	cyclo[N(Me)Bmt(E)-Thr-Sar-Mle-Val-Mle-Ala-Dal-Mle-Mle-Mva]	C${}_{62}$H${}_{111}$N${}_{11}$O${}_{13}$	59787-61-0
CsE	cyclo[N(Me)Bmt(E)-Abu-Sar-Mle-Val-Mle-Ala-Dal-Mle-Mle-Val]	C${}_{61}$H${}_{109}$N${}_{11}$O${}_{12}$	63798–73-2
CsH	cyclo[N(Me)Bmt(E)-Abu-Sar-Mle-Val-Mle-Ala-Dal-Mle-Mle-N(Me)dVal]	C${}_{62}$H${}_{111}$N${}_{11}$O${}_{12}$	83602-39-5
CsL	cyclo[Bmt(E)-Abu-Sar-Mle-Val-Mle-Ala-Dal-Mle-Mle-Mva]	C${}_{61}$H${}_{109}$N${}_{11}$O${}_{12}$	108027-39-0

**Table 2:** ${}^{3}J$(NH–Hα) couplings in cyclosporins (Hz), measured from NMR spectra and calculated from MD trajectories. Numbers (1) and (2) stand for the uni- and bimodal angle distributions in the simulation. Residue Xxx2 is Thr or Abu in different cyclosporins (see Table 1). The ${}^{1}$H NMR spectrum of cyclosporin L shows two coexisting conformers.

	Bmt1	Xxx2	Val5	Ala7	Dal8	Val11
CsC		9.7	8.9	7.0	7.2	
		5.63 (1)	8.49 (1)	7.88 (1)	7.07 (1)	
CsE		9.8	8.4	9.1	6.2	9.7
		7.94 (2)	8.57 (2)	8.18 (2)	7.62 (2)	8.25 (2)
CsH		5.8	7.2; <5.2	n/d	8.1	
		7.61 (1)	6.45 (2)	6.68 (1)	6.70 (1)	
CsL	7.5	9.9	8.6	7.3	8.4	
		10.0	8.7	6.9	n/d	
	8.72 (1)	5.78 (2)	8.02(2)	7.61 (2)	7.2 (1)	

**Table 3:** Appearance of hydrogen bonds during the simulation. The left column shows the interacting atoms (O is the carboxyl oxigen; H, amide proton; Hg and Og are atoms in the γ-positions). The table shows which H-bonds are stable: relative duration of their existence (%) during the simulation and the length of the longest detected existence period (ns, in parentheses).

	CsC	CsL	CsH	CsE
Bmt1(H)-Mle10(O)	–	100%	–	–
ine Bmt1(Og1)-Ala7(H)	81% (14.2)			
ine Bmt1(Hg1)-Bmt1(O)	97% (18.7)	91% (38)	44% (12.5)	19% (19.9)
ine Val5(H)-Sar3(O)	96% (21.4)		47% (13.4)	100%
ine Bmt1(Hg1)-Val5(O)	13% (1.2)	43% (8)		
ine Thr2(Hg1)-Thr2(O)	58% (2.4)	–	–	–
ine Thr2(H)-Mle6(O)				88.8% (19)
ine Ala7(H)-Bmt1(Og1)		50% (18)		
ine Dal8(O)-Val11(H)				51% (5.8)
ine Dal8(H)-Val11(O)		76%		58% (9.6)

## Supplementary Materials

The following supporting information can be downloaded at: https://www.mdpi.com/article/10.3390/membranes13020196/s1, Figure S1: Distance from the peptide’s center of geometry to the micelle’s center; Figure S2: RMSD of atom positions related to the starting frame; Figure S3: Distributions of the proton–oxygen distances in pairs which can form hydrogen bonds in cyclosporin C; Figure S4: Time dependencies of the proton–oxygen distances in pairs which can form hydrogen bonds in cyclosporin L (plots a, c). Other plots (b, d–f) show frequency distribution of the distances met over the MD trajectory. Existance of a narrow peak at d 0.2 nm sorresponds to the states with existing H-bond; relative height of the peaks in multimodal distributions allow estimating the relative populations of the conformations having the particular H-bond; Figure S5: Frequency distribution of the distances met over the MD trajectory in two independent simulations; Figure S6: Distributions of the proton–oxygen distances in pairs which can form hydrogen bonds in cyclosporin E; Figure S7: NOESY spectrum of cyclocporin C (NH and Hα signals); Figure S8: NOESY spectrum of cyclocporin E (NH and Hα signals); Figure S9: Cyclosporin L and the DPC micelle. The peptide is shown in yellow (residues Bmt1–Ala7) and pink (Dal8–Mva11, the turn region); Figure S10: Cyclosporin E and the DPC micelle. The peptide is shown in black and green (residue Bmt1). Cross-eyed stereo; Figure S11: Cyclosporin C and the DPC micelle before and after the NPT simulation. The peptide is shown in black and green (residue Bmt1).

## Abbreviations

The following abbreviations are used in this manuscript:

NMR	Nuclear magnetic resonance
MD	Molecular dynamics
DPC	Dodecylphosphocholine
SDS	Sodium dodecylsulphate
