# Supplementary material for: Binding of Different Cyclosporin Variants to Micelles Evidenced by NMR and MD Simulations
Source: Membranes (Basel). 2023 Feb 5;13(2):196. doi: 10.3390/membranes13020196 (PMC9965255; doi:10.3390/membranes13020196)
Supplement: Supplementary file 1 [file membranes-13-00196-s001.zip › membranes-2100388-supplementary.pdf]

Binding of Different Cyclosporin Variants to Micelles Evidenced by NMR and MD Simulations

Supplementary Info

P.P. Kobchikova*, S.V. Efimov, and V.V. Klochkov

Institute of Physics, Kazan Federal University, 18 Kremlevskaya, Kazan 420008, Russia;
pollymoon@yandex.ru

1 Interpretation of Molecular Dynamics Data

The main attention in this work is paid to three cyclosporins: C, E and L. Some details about the systems were prepared for the sumulation: (1) CsC was in the cubic box with the initial side of 5.2 nm (before the NVT step), 47 DPC unimers and 4983 water molecules gave $197 + 47 * 29 + 3 * 4983 = 16509$ atoms total, computing rate was 19.824 ns/day; (2) CsE – size 5.0 nm, $193 + 29 * 52 + 3 * 3231 = 11394$ atoms, 16.389 ns/day; (3) CsL – size 5.2 nm, $193 + 51 * 29 + 4 * 3792 = 16840$ atoms, 18.893 ns/day.

Basic characteristics of the simulated system: plots of the distance from the the peptide ring to the micelle center (Fig. S1) and RMSD of backbone heavy atoms positions (Fig. S2).

Additional MD data: time behaviour and distribution of interatomic distances. Distance between an NH (or OH) proton and a CO (or OH) oxygen was observed as one of the criterions of hydrogen bond formation. Evidently, in many cases the H-bond appears and breaks from time to time, and the duration of the periods corresponding to either state can vary in a wide range. Frequency analysis is a convenient way to estimate the relative probability of different states met along the simulated MD trajectory.

Figure S3 shows distribution of interatomic distances for CsC. Here you can note that the intraresidue H-bond is formed in threonine-based amino acid Bmt1 (plot **a**), but in Thr2 it seems to be longer and weaker (**b**). The distance Ala7(H)–Bmt1(Oγ1) (**c**) gives the example of stable H-bond, while atoms Bmt1(Hγ1) and Val5(O) (plot **d**) are rarely close enough to have a chance to form an H-bond.

Figure S4 shows the time behaviour and distribution of distances in CsL in a way similar to Fig. S3. For example, the distance Dal8(H)–Mva11(O) in CsL (Figure S4**c**) is short for first 38 ns (except for several short events when it suddenly increases to nearly 0.5 nm), and also from 52 to 56 ns. So, we can expect that the corresponding H-bond exists for at least 70% time (from the simulated total 80 ns), and the plot in Figure S4**d** gives the numeric proportion of 350:100 (peak heights). Solid lines in the plot were obtained by Gaussian fitting.

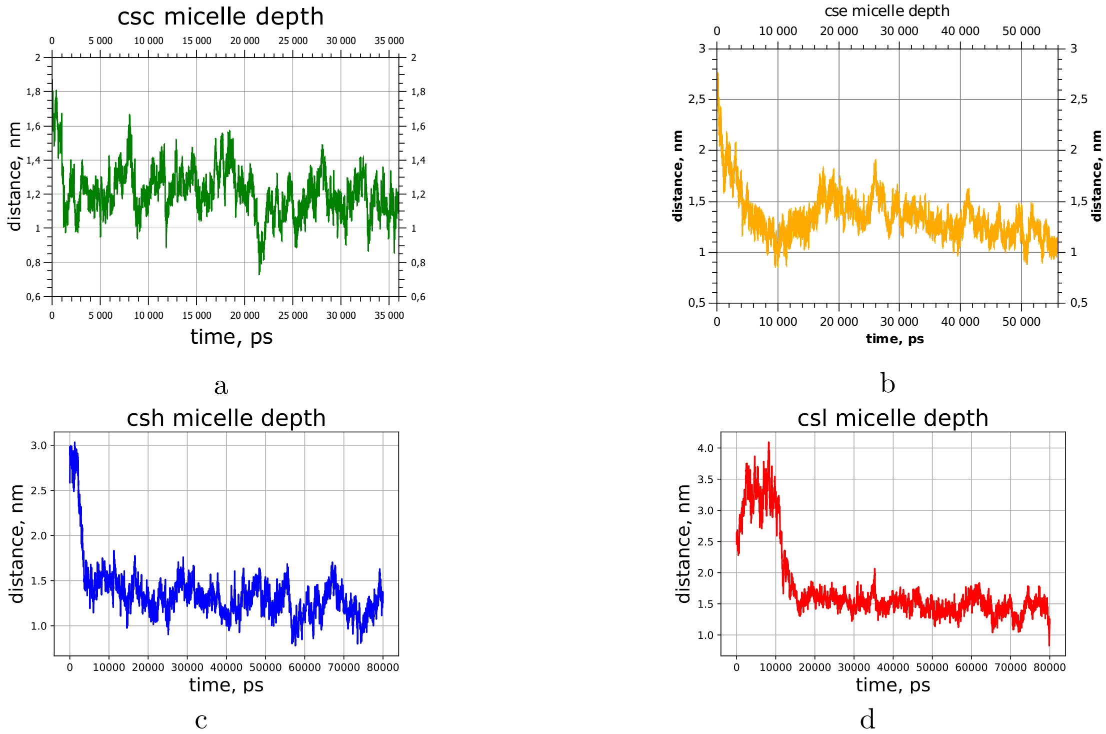

Figure S1: Distance from the peptide's center of geometry to the micelle's center.

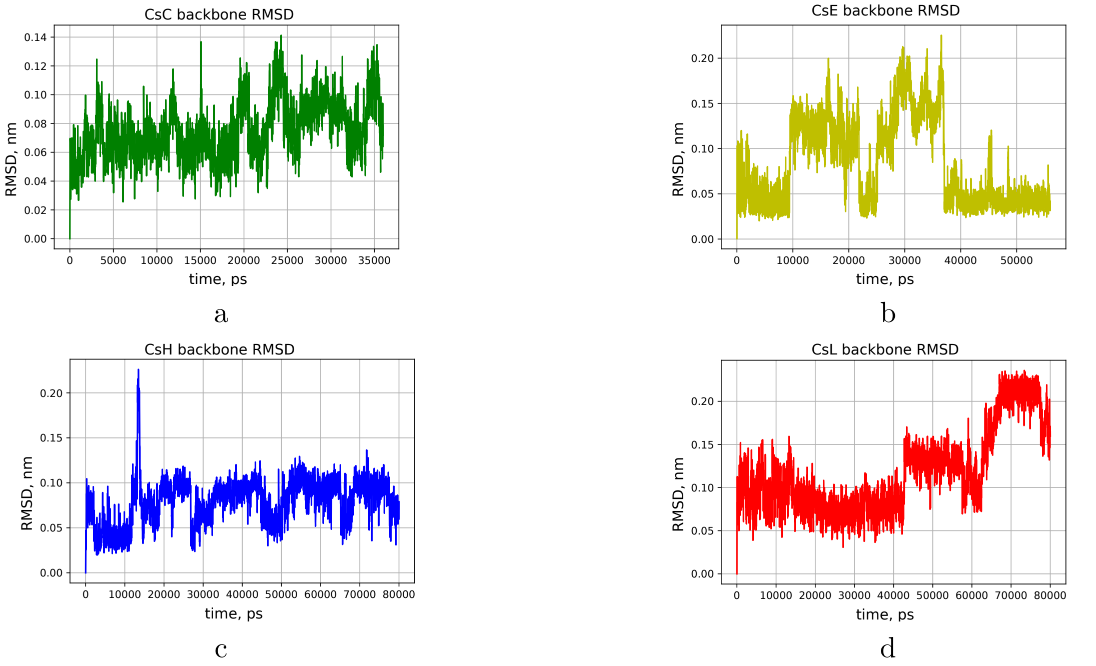

Figure S2: RMSD of atom positions related to the starting frame.

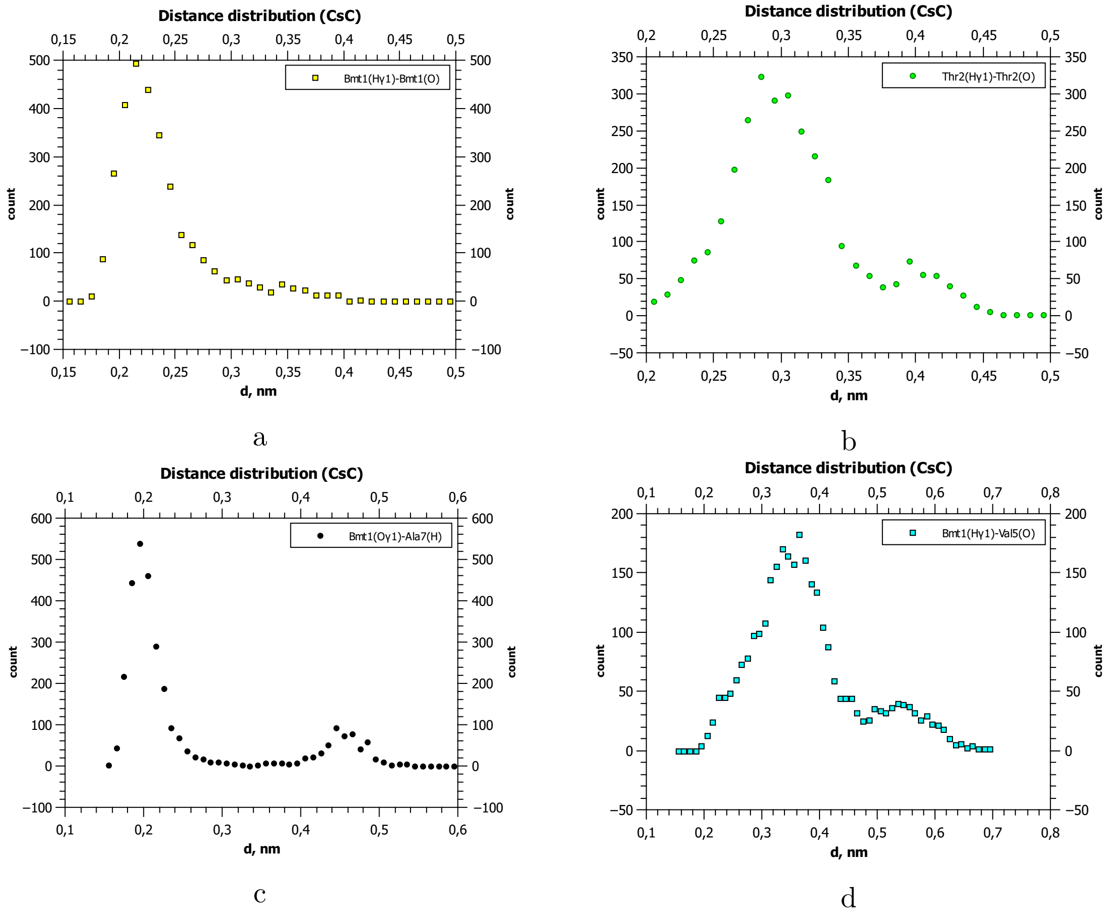

Figure S3: Distributions of the proton–oxygen distances in pairs which can form hydrogen bonds in cyclosporin C.

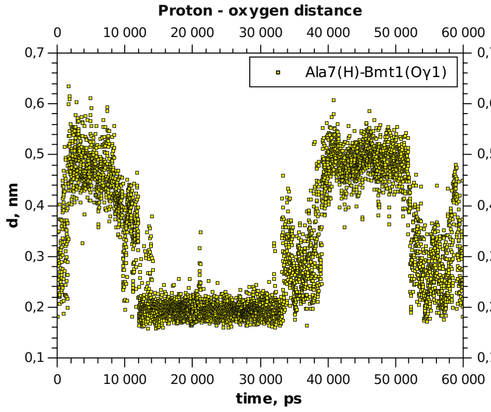

a

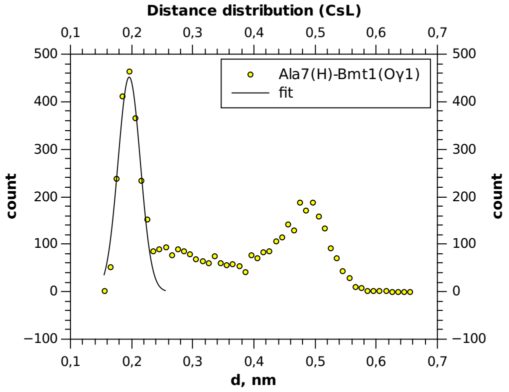

b

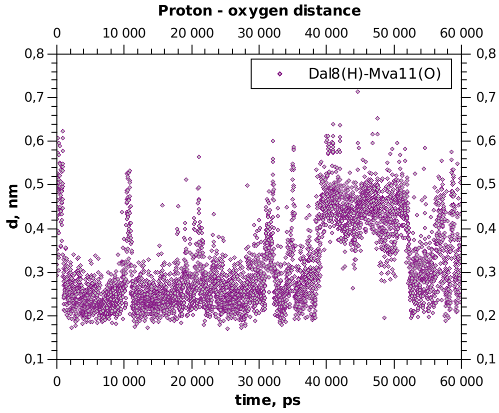

c

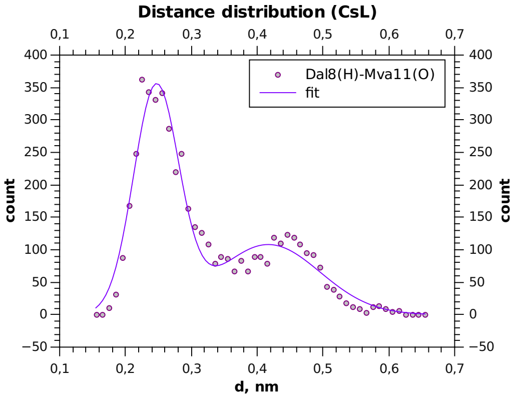

d

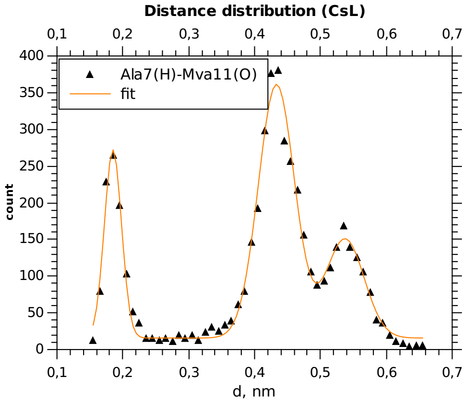

e

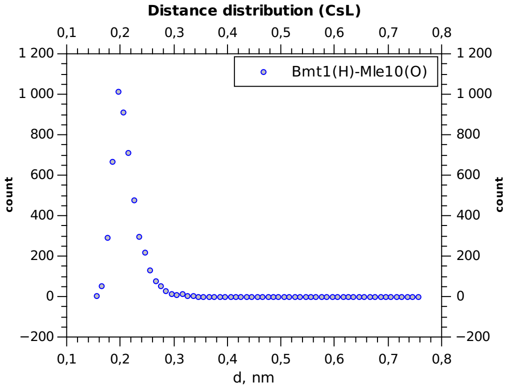

f

Figure S4: Time dependencies of the proton–oxygen distances in pairs which can form hydrogen bonds in cyclosporin L (plots **a**, **c**). Other plots (**b**, **d**–**f**) show frequency distribution of the distances met over the MD trajectory. Existance of a narrow peak at $d \sim 0.2$ nm sorresponds to the states with existing H-bond; relative height of the peaks in multimodal distributions allow estimating the relative populations of the conformations having the particular H-bond.

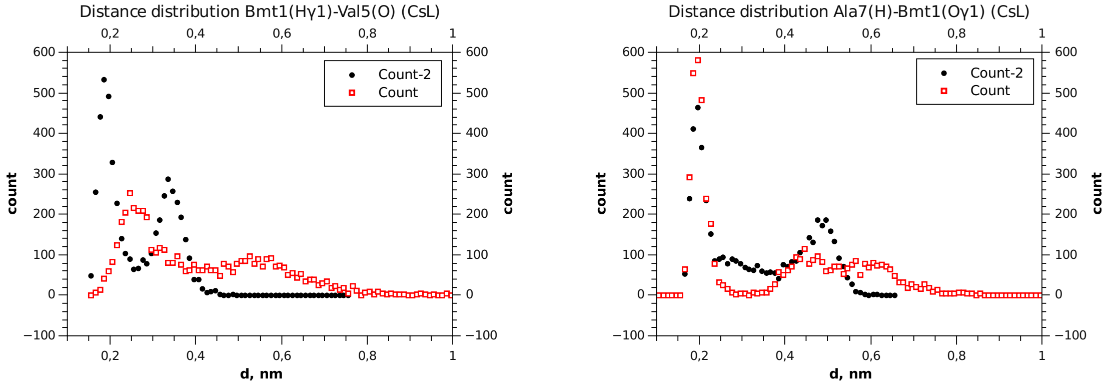

a

b

Figure S5: Frequency distribution of the distances met over the MD trajectory in two independent simulations.

In the case of CsL two independent simulations were performed with different initial atom dispositions and velocities. In both cases the peptide ring was finally attached to the micellar surface. Formation of H-bonds in atom pairs Bmt1(Hγ1)–Val5(CO) and Ala7(NH)–Bmt1(Oγ1) was also observed in these two runs, while formation of H-bonds like Bmt1(Hγ1)–Bmt1(CO) or Bmt1(Hγ1)–Mle10(CO) occurred only in one of the simulations. Evidently, long time is needed to test all possible variants of intramolecular hydrogen bonding; even in the case of well-reproduced distances, the proportion of simulation frames when the distance $d \sim 0.2$ nm and $d > 0.25$ nm may differ noticeably, as shown here in Figures S5(a, b).

Figure S6 shows distribution of interatomic distances for CsE. Note that after first 30 ns of simulation the character of the behaviour suddenly changes: this can be seen in the distances Bmt1(Hγ1)–Bmt1(O), Abu2(H)–Mle6(O) or Dal8(H)–Val11(O) (plots **a**, **b**, **g**). However, the situation in the pairs Abu2(H)–Val11(O) (H-bond mostly absent) and Val5(H)-Sar3(O) (H-bond mostly present) remains nearly the same before and after that moment.

2 NMR Data

NOESY spectra of cyclosporins in complex with DPC micelles (700 MHz). The spectra were recorded with water suppression using pulse sequences noesyphpr or noesygpph19, and then visualised and assigned using Sparky programme. Blue labels in Figs. S7 and S8 designate cross-peaks between atoms in different amino acids; diagonal peaks and intraresidue cross-peaks are labelled in black. Note the far contact Bmt1–Mle6 in CsE (red label in Fig. S8).

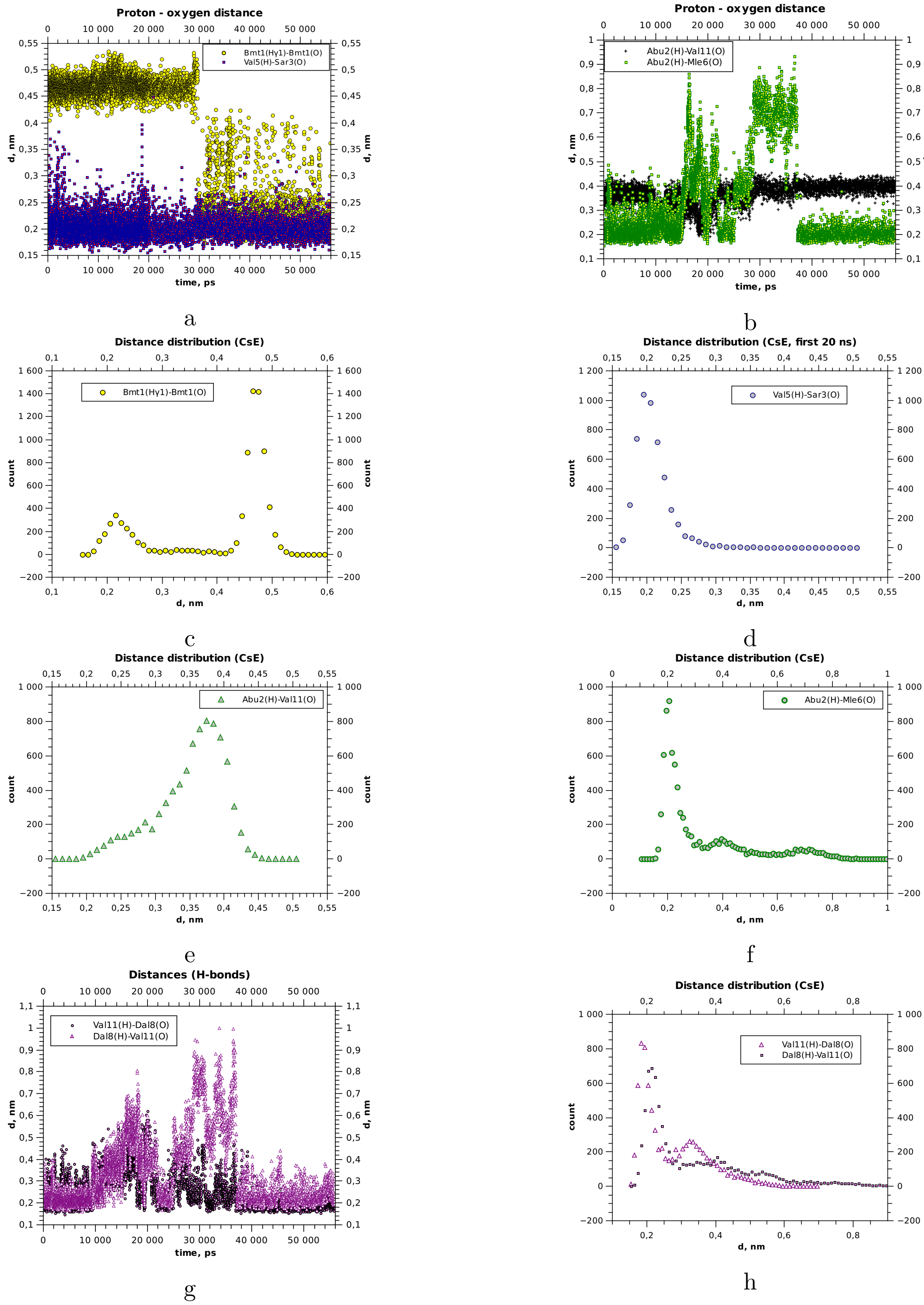

Figure S6: Distributions of the proton–oxygen distances in pairs which can form hydrogen bonds in cyclosporin E.

3 Images of the Simulated Systems

Several snapshots of the MD trajectory show how the CsL molecule is located with respect to the DPC micelle (Fig. S9). Once trapped to the surface, the molecule remain there, but does not penetrate deeped in the course of the performed simulation. The next Fig. S10 demonstrates the disposition of CsE at the beginning and in the end of the trajectory (moments of 36 and 56 ns). The peptide molecule slightly rotates in its place but remains nearly at the same depth. Finally, Fig. S11 shows the initial and final snapshots of cyclosporin C.

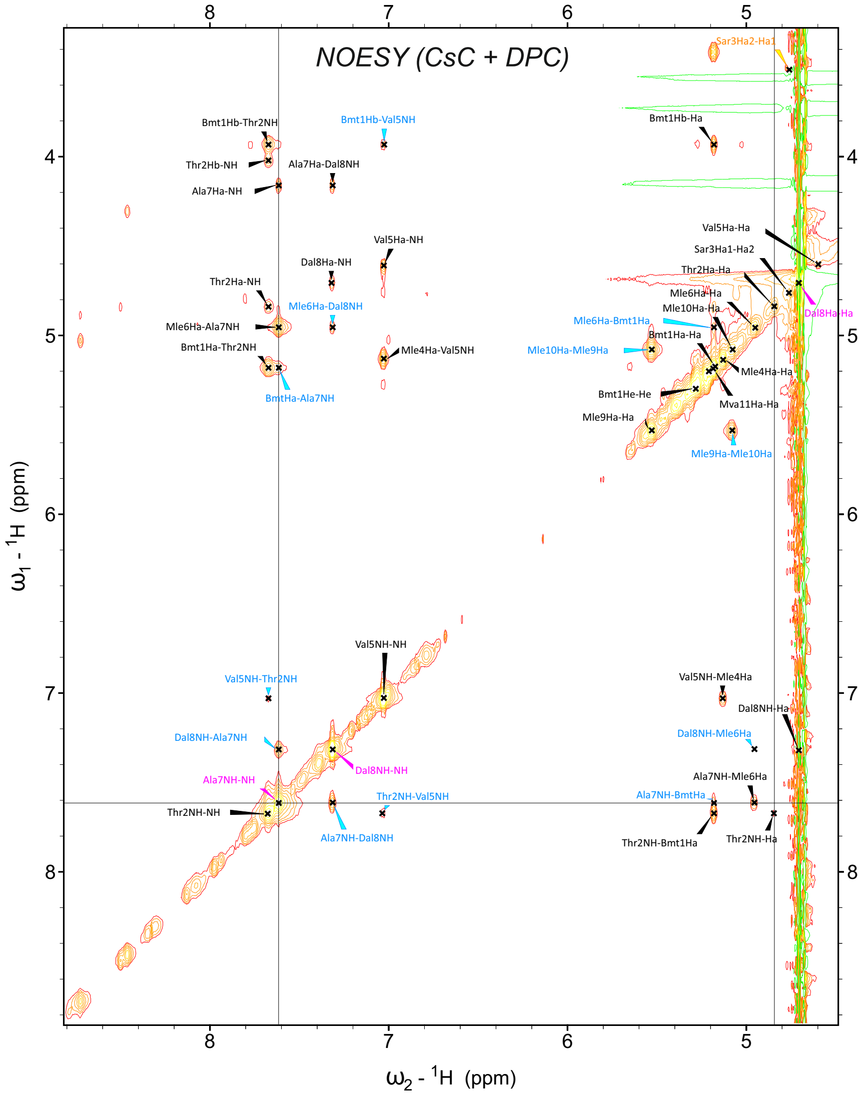

Figure S7: NOESY spectrum of cyclocporin C (NH and Hα signals).

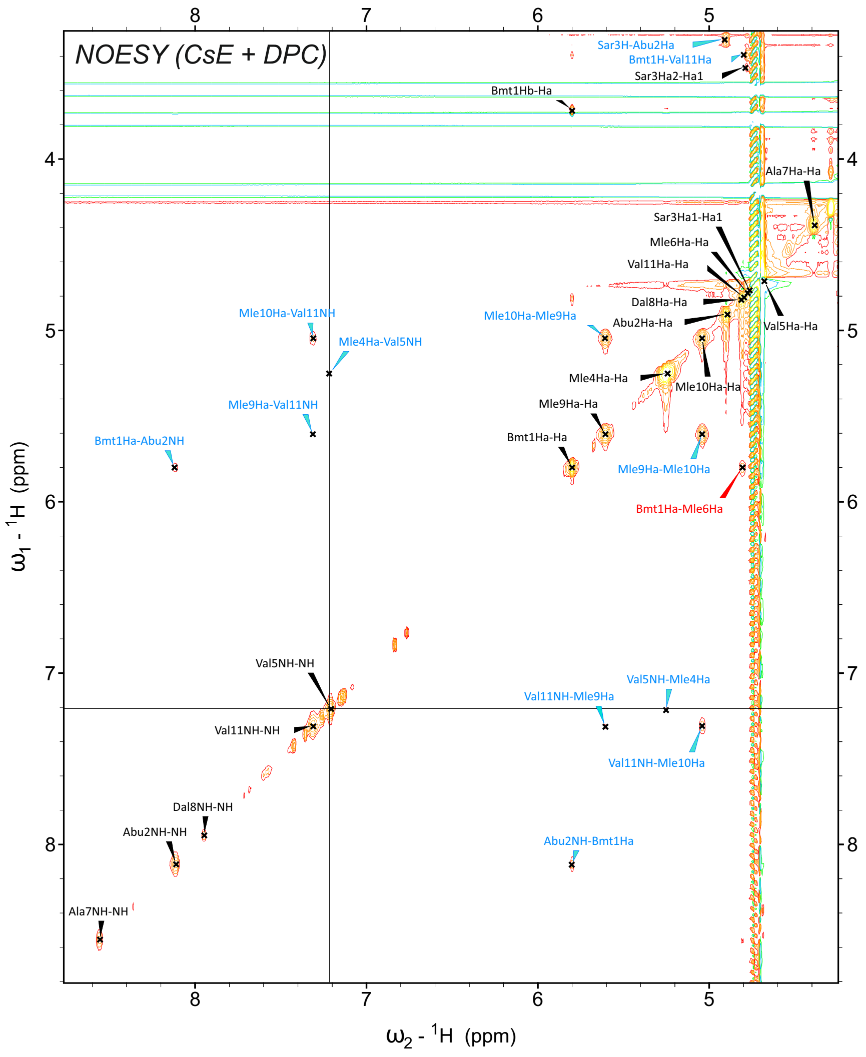

Figure S8: NOESY spectrum of cyclocporin E (NH and Hα signals).

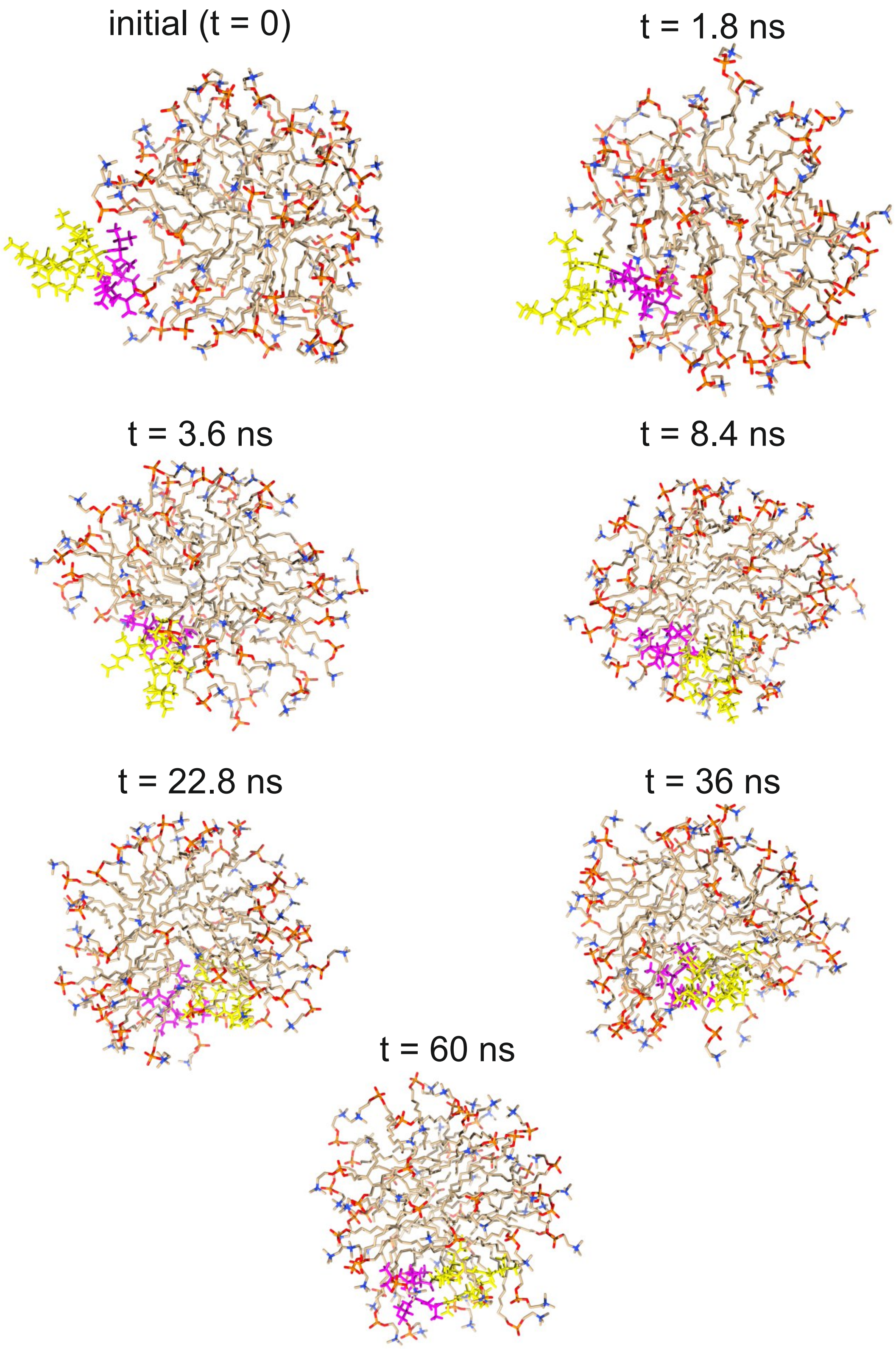

Figure S9: Cyclosporin L and the DPC micelle. The peptide is shown in yellow (residues Bmt1–Ala7) and pink (Dal8–Mva11, the turn region).

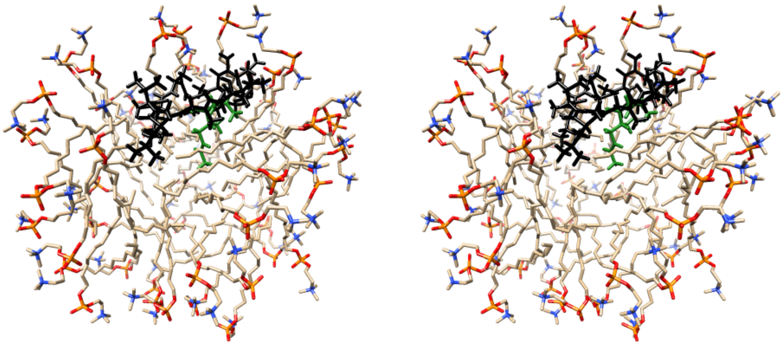

t = 26 ns

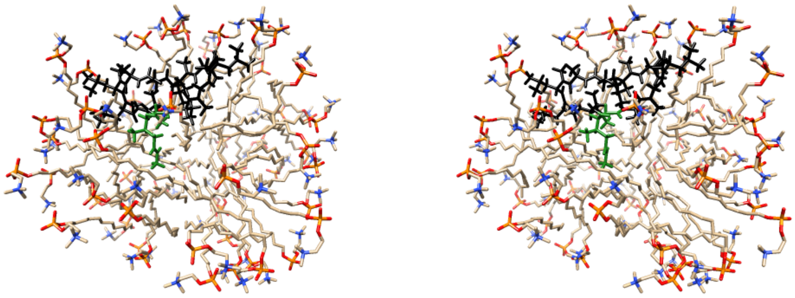

t = 56 ns

Figure S10: Cyclosporin E and the DPC micelle. The peptide is shown in black and green (residue Bmt1). Cross-eyed stereo.

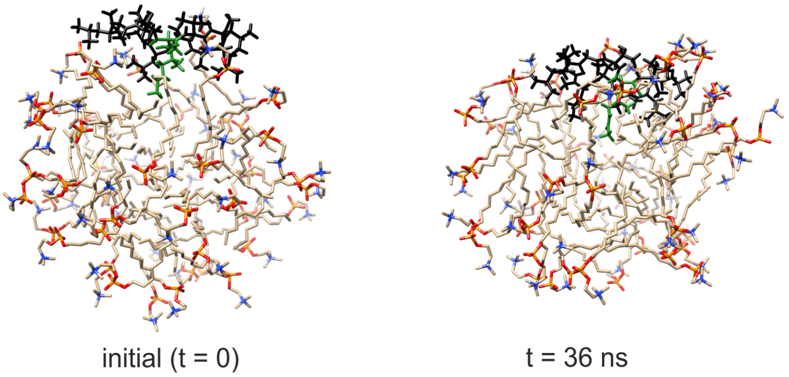

Figure S11: Cyclosporin C and the DPC micelle before and after the NPT simulation. The peptide is shown in black and green (residue Bmt1).
